# The genome-wide transcriptional consequences of the nullisomic-tetrasomic stocks for homoeologous group 7 in bread wheat

**DOI:** 10.1186/s12864-018-5421-3

**Published:** 2019-01-10

**Authors:** Rongzhi Zhang, Shuaifeng Geng, Zhengrui Qin, Zongxiang Tang, Cheng Liu, Dongfeng Liu, Guoqi Song, Yulian Li, Shujuan Zhang, Wei Li, Jie Gao, Xiaodong Han, Genying Li

**Affiliations:** 1 Key Laboratory of Wheat Biology & Genetic Improvement on North Yellow & Huai River Valley, Ministry of Agriculture, National Engineering Laboratory for Wheat & Maize, Institute of Crop Science, Shandong Academy of Agricultural Sciences (SAAS), #202, Road of Gongyebei, Jinan, 250100 China; 2 Institute of Crop Science, Chinese Academy of Agricultural Sciences, Beijing, 100081 China; 3 Agronomy College, Sichuan Agricultural University, Wenjiang, Chengdu, 610054 China; 4 Key Laboratory of Plant Molecular Physiology, Institute of Botany, Chinese Academy of Sciences, Beijing, 100093 China

**Keywords:** Gene expression, Dosage deletion, Dosage compensation, *Triticum aestivum L*, Nullisomic-tetrasomic stocks

## Abstract

**Background:**

Hexaploid bread wheat (*Triticum aestivum L*) arose by two polyploidisation events from three diploid species with homoeologous genomes. Nullisomic-tetrasomic (nulli-tetra or NT) lines are aneuploid wheat plants lacking two and adding two of six homoeologous chromosomes. These plants can grow normally, but with significantly morphological variations because the adding two chromosomes or the remaining four chromosomes compensate for those absent. Despite these interesting phenomena, detailed molecular mechanisms underlying dosage deletion and compensation in these useful genetic materials have not been determined.

**Results:**

By sequencing the transcriptomes of leaves in two-week-old seedlings, we showed that the profiles of differentially expressed genes between NT stocks for homoeologous group 7 and the parent hexaploid Chinese Spring (CS) occurred throughout the whole genome with a subgenome and chromosome preference. The deletion effect of nulli-chromosomes was compensated partly by the tetra-chromosomes via the dose level of expressed genes, according to the types of homoeologous genes. The functions of differentially regulated genes primarily focused on carbon metabolic process, photosynthesis process, hormone metabolism, and responding to stimulus, and etc., which might be related to the defective phenotypes that included reductions in plant height, flag leaf length, spikelet number, and kernels per spike.

**Conclusions:**

The perturbation of the expression levels of transcriptional genes among the NT stocks for homoeologous group 7 demonstrated the gene dosage effect of the subgenome at the genome-wide level. The gene dosage deletion and compensation can be used as a model to elucidate the functions of the subgenomes in modern polyploid plants.

**Electronic supplementary material:**

The online version of this article (10.1186/s12864-018-5421-3) contains supplementary material, which is available to authorized users.

## Background

Chromosomes are partitioned equally during cell division for most eukaryotic organisms [1]. However, aneuploid organisms may result from aberrant segregation events. In humans and other animals, aneuploidy always leads to abortions and developmental disabilities, such as Down’s syndrome with a trisomy of chromosome 21 in humans, and is associated with solid tumours [2]. The reasons remain very unclear for the higher tolerance of plants for aneuploidy than that of animals. The failure to equally partition the multiple chromosome sets at meiosis causes aneuploid individuals. This phenomenon frequently appears spontaneously within polyploid plant populations [3]. For the aneuploid syndromes, the relationships between phenotypes and karyotypes have been investigated in populations of aneuploid *Arabidopsis thaliana* [4]. The deficient phenotypic traits are observed in the branching-type, curly-leaves, empty-axils, meristematic reversions, and irregular-spacing, and etc. The dose of specific chromosome types contributes to certain traits, and the chromosomal effects can be additive. The aberrant gene expression of aneuploidy always drives the whole-genome dosage imbalance, which may cause additive effects of the phenotypic deficiencies [5]. In hexaploid wheat, recently a study using the aneuploid wheat including monosomic 1A/1B/1D/2A, trisomic 1A/2A, tetrasomic 1A, nullisomic 1A, nulllisomic 1A and tetrasomic 1B, and nulllisomic 1A and trisomic 1B, showed that chromosomes and subgenomes in hexaploid wheat are unequal in their responses to the aneuploid condition. These aneuploid wheat also behaved the abnormal variations in typical phenotypic traits including reduced plant height, higher spikelet density, reduced seed-setting and shorter spike length [6].

Allohexaploid wheat arose by two polyploidisation events from three diploid species with homoeologous genomes. The first hybridisation event occurred in the genomes of two diploid species, i.e.*, Triticum urartu* (AA, 2n = 2x = 14) and *Aegilops speltoides* (SS, 2n = 2x = 14), forming the allotetraploid wheat, *Triticum turgidum* (AABB, 2n = 4x = 28). In the second hybridisation event, another diploid grass species, *Aegilops tauschii* (DD, 2n = 2x = 14), was introduced into the allotetraploid wheat, producing the ancestral allohexaploid *Triticum aestivum* (AABBDD, 2n = 6x = 42) [7, 8], which possesses three homoeologous sets of seven chromosomes, i.e.*,* subgenome A, subgenome B and subgenome D. Unlike the paleopolyploid maize and soybean, no transcriptional dominance was observed among the three sets of subgenomes at the genome-wide level in the modern wheat [8]. However, the molecular mechanism of how these subgenomes work together remains unclear. Chinese Spring (CS) is a cultivated bread wheat in which many aneuploid stocks including nullisomic-tetrasomic (nulli-tetra or NT) stocks in the mid-twentieth century were developed by E. R. Sears 1952 [9, 10]. Based on the resemblances between different nullisomic stocks, in the homoeologous groups 1–7 of three subgenomes, all of the 42 possible NT combinations within groups have been synthesized [9, 10]. Within these groups, each tetrasome shows the ability to compensate for either of the other two nullisomes in some degree, which shows some superiority over the simple nullisomic stocks. In some cases, the compensation was nearly complete. Within groups 1, 3, and 7, all the NT stocks were observed as reasonably normal plants, which demonstrates the close genetic relation for the three chromosomes in each of these groups.

According to the characteristics of chromosomes, these stocks are currently primarily used for localisation of genes on chromosomes. Although one study [6] showed the deletion and compensation dosage effect using the first and second groups of aneuploid wheat, study of the detail molecular mechanisms of gene dosage balance with different subgenome karyotypes in the NT stocks for group 7 has not been conducted. The tetrasomes could possibly compensate for the nullisomes by supplying the necessary gene products and permit normal growth, possibly with significantly morphological variation in plant height, flag leaf length, spikelet number, or kernels per spike, and so on [6]. Because the deficient phenotypes of NT stocks are presumably due to disturbances of gene expression, the investigation of the molecular mechanisms requires an understanding of the transcriptome differences between the NT stocks and CS. Recently, the availability of genome sequences of CS offered the opportunity to gain insights into the dosage compensation mechanisms for the NT stocks. In this study, we chose the NT stocks for group 7, and used RNA sequencing to investigate how the tetrasomes compensated for the nullisomes at the transcriptome level.

## Methods

### Plant material

The Wheat Genetics Resource Center (WGRC, http://www.k-state.edu/wgrc/) provided the seeds of the NT stocks for group 7: N7AT7B, N7AT7D, N7BT7A, N7BT7D, N7DT7A and N7DT7B. We planted these seeds in the field and only harvested those spikes bagged before flowering. Then, according to published methods [11, 12], for these reproduced seeds, we first verified the karyotypes of chromosomes by cytogenetic identification with Fluorescence In Situ Hybridisation (FISH) on the metaphase of the NT stocks using Oligo-pTa535-1 (red) and Oligo-pSc119.2 (green) as probes (Additional file 1: Figure S1). Then, we planted the verified seeds with the correct chromosome karyotypes in the greenhouse at 16/8 h light/dark.

### RNA isolation and sequencing library construction

The leaves of two-week-old seedlings were harvested for RNA extraction and sequencing. Total RNAs were isolated and the quality was assayed as previously described [13]. The mRNA libraries were constructed according to the standard protocol of the manufacturer (Illumina), and the sequencing was performed according to the instructions of the manufacturer with a Hiseq2000 machine (Illumina). The seven RNA-seq datasets including N7AT7B, N7AT7D, N7BT7A, N7BT7D, N7DT7A, N7DT7B and CS were deposited in the NCBI SRA database with the accession number SRR5140981, SRR5140982, SRR5140983, SRR5140984, SRR5140985, SRR5140986 and SRR5140987, respectively.

### Reads mapping to the genome and gene expression analysis

Reference genome sequences of CS (version 2) were downloaded from the IWGSC website. Reads were then mapped to reference sequences without mismatch using the Tophat2 program [14]. To precisely obtain the gene expression level from the three subgenomes we used only uniquely mapped reads to calculate the RPKM value, which was done by perl scripts. This step removed quite amount of reads but was required in accurately estimate gene expression levels because one read can not be used twice. Then, we used those uniquely mapped reads and the length of the gene model as RPKM value using Cufflinks program following the transcript expression analysis protocols [15]. Differential analysis of RNA-seq data without biological replicates was performed using DEGseq program which uses a MA-plot-based method with random sampling model to simulate technical variations [16]. To guarantee accuracy, differentially expressed genes with FDR less than 0.001 were selected to further analysis in the next step. The detailed flow chart was presented in Additional file 2: Figure S2.

### Gene ontology enrichment analysis for the significantly differentially expressed genes

The GO (Gene Ontology) term annotation of wheat genes is available in the IWGSC website. The functional enrichment of significantly expressed genes was performed by the BINGO program with reference to the CS genome [17]. The hypergeometric test method was applied to calculate the enrichment *P-value* (*P-value* < 0.05), and the FDR correction of the Benjamini and Hochberg multiple hypothesis testing method was used to reduce the false negatives with FDR < 0.05 [18].

## Results

### Deficient phenotypes determined by chromosome patterns

**Fig. 1:**
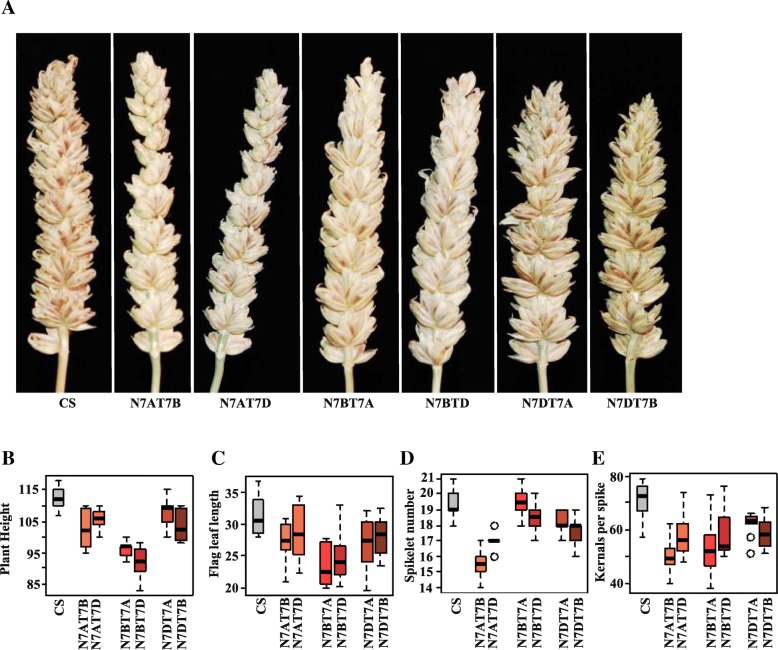
Phenotypes of the NT stocks for group 7 and CS in Spike (**a**), plant height (**b**), flag leaf length (**c**), spikelet number (**d**), and kernels in per spike (**e**)

To compare the phenotypic traits between the NT stocks for group 7 and euploid CS, several phenotypical characters were investigated (Fig. 1a-e). Reductions in plant height, flag leaf length, spikelet number, or kernels per spike were observed in these NT stocks (Fig. 1b-e). In N7AT7B and N7AT7D stocks, the increased space between the spikelets (Fig. 1a) led to fewer number of spikelets (Fig. 1d) and contributed to fewer kernels in per spike (Fig. 1e). In N7BT7A and N7BT7D stocks, although the spikelet number was similar to that of CS (Fig. 1d), the infertility of the top spikelets (Fig. 1a) led to fewer kernels per spike (Fig. 1e). In N7DT7A and N7DT7B stocks, the reduction in spike length (Fig. 1a), which led to reduction in spike number (Fig. 1d), and the infertility of the top spikelets (Fig. 1a) contributed to fewer kernels in per spike (Fig. 1e). Although these NT stocks exhibited deficient phenotypes, all were fertile, which indicated that the consequences of nulli-chromosome dosage imbalance might be buffered by the replaced homoeologous tetra-chromosome or other homologous genes derived the paleoploid copies. Additionally, the similar deficiently phenotypic traits that corresponded to similar chromosome deletion karyotypes might indicate that the deletion dosage of one pair of chromosomes can not be completely compensated by their homoeologous chromosomes, which might be due to the functional divergence of the homoeologous genes.

### Gene expression pattern followed the pattern of the chromosome karyotype

**Fig. 2:**
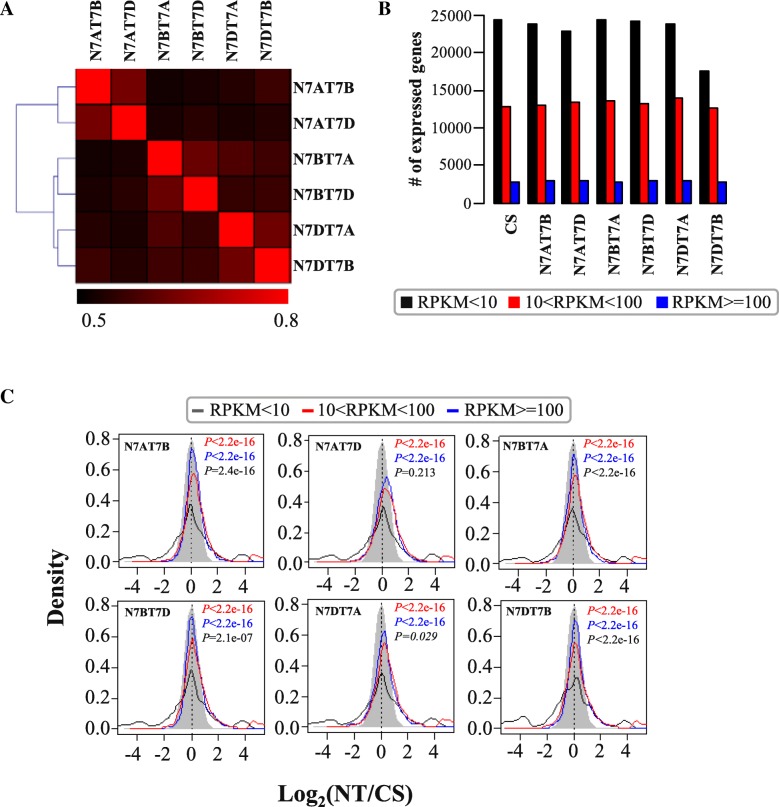
Gene expression profiles in the NT stocks for group 7. **a** The correlation of gene expression among the NT stocks for group 7. **b** The distribution of the lowly (RPKM < 10, black rectangles), mediumly (10 < = RPKM < 100, red rectangles), and highly (RPKM > = 100, blue rectangles) expressed genes. **c** The density distribution of fold change (log_2_(NT/CS)) of gene expression for the lowly (black lines), mediumly (red lines), and highly (blue lines) expressed genes in the NT stocks for group 7 compared with CS. The grey background represented the model normalisation distribution

Different types of chromosome karyotypes exhibited differently phenotypic traits, which should be defined as the expressed gene dosage effect. Thus, to investigate the differences in the gene expression profiles among the NT stocks for group 7 and euploid CS, seven RNA-seq libraries were constructed from the leaves of two-week-old seedlings and sequenced. Approximately 11–12 million reads were generated by RNA sequencing, and ~ 8–9 million reads were perfectly mapped to the reference genomes of CS (Table 1) by Tophat software. To precisely distinguish the gene expression in subgenome A, subgenome B, and subgenome D, those reads that were mapped to at least two sets of subgenomes were removed. Approximately 0.8–1 million reads were only uniquely mapped to subgenome A, subgenome B, or subgenome D (Additional file 3: Table S1). Then, using these one-subgenome-specific reads, the gene expression level was calculated with the Cufflink program (Additional file 2: Figure S2). A total of 32–40 thousand genes were expressed (Additional file 4: Table S2). Nullisomic stocks with the same chromosome pair missing were more similar in gene expression levels, such as those between N7AT7B and N7AT7D, among which two 7A were replaced by two 7B or two 7D as shown on the left side of Fig. 2a. Similar clustering was found for N7BT7A and N7BT7D stocks, as well as N7DT7A and N7DT7B stocks. Such a close correlation may also be reflected on their spike phenotypes as can be seen in Fig. 1 where spikes of N7AT7B and N7AT7D were more similar and spikes of N7BT7A and N7BT7D were more similar and so were N7DT7A and N7DT7B. Thus, it may suggest that similar karyotypes of chromosomes with similar gene expression profiles might be associated with their similar phenotypical traits.

**Table 1:** Total mapping information of the transcriptomes referred to the CS genome without mismatch in the NT stocks for group 7 and CS^a^

Lines	Raw reads	Mapped reads
N7AT7B	11,983,730	8,561,473 (71.44%)
N7AT7D	11,690,116	8,611,236 (73.66%)
N7BT7A	11,685,574	8,659,035 (74.10%)
N7BT7D	11,632,836	8,469,241 (72.80%)
N7DT7A	11,929,967	8,855,439 (74.22%)
N7DT7A	11,486,736	8,681,326 (75.58%)
CS	12,279,563	9,098,871 (74.10%)

^a^Chinese Spring genome version 2

To further understand the gene differential expression profiles, we classified the expressed genes into three categories: lowly expressed genes (LEG, RPKM < 10), mediumly expressed genes (MEG, 10 < = RPKM < 100), and highly expressed genes (HEG, RPKM > = 100). Totally, 17–24, 12–14, and more than two thousand genes were identified at the low, medium, and high expression level, respectively (Additional file 4: Table S2 and Fig. 2b). Most of genes had low and medium expression levels in the NT stocks. With the expected model of normal distribution, we compared the fold-change (log_2_(NT/CS)) distribution of gene expression for each category and found that the medium and high expression genes contributed substantially to up-regulation in the NT stocks compared with CS (*P < 2.2e-16*; Fig. 2c). By contrast, the lowly expressed genes were nearly equal for the up- and down-regulated genes. The overall gene expression dosage patterns of all NT stocks were similar in terms of total expression gene numbers and their differential expression patterns as shown in Fig. 2b-c. Thus, the analogous dosage variation of expressed genes indicated a similar deletion and compensation response mechanism for the multiploidy.

### Highly expressed genes as dosage-sensitive genes contributed to the dosage compensation of the chromosomes

**Fig. 3:**
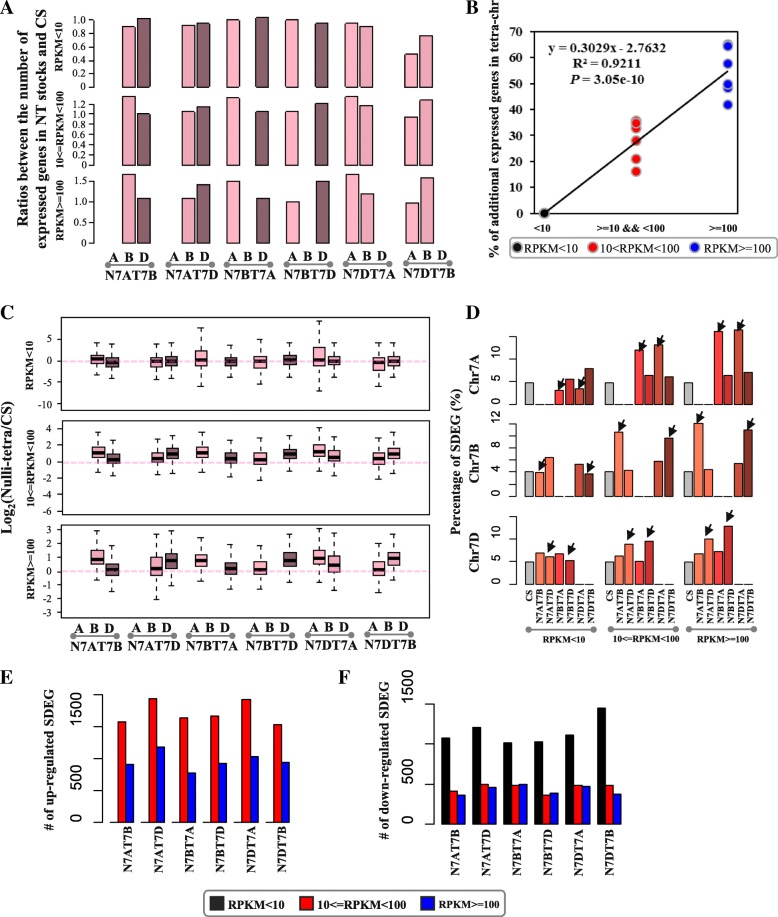
The distribution of ratios of gene number (NT/CS; **a**) and gene expression level (log_2_(NT/CS); **c**) for the lowly, mediumly, and highly expressed genes in the NT stocks for group 7 compared with CS. The pink, light pink, and dark pink rectangles represented the subgenome A, subgenome B, and subgenome D, respectively. **b** Correlation between the expression level and the percentage of up-regulated genes. **d** The preference of proportion distribution of SDEGs on the tetra-chromosome 7A, 7B, and 7D. **e**-**f** The up- (**e**) and down- (**f**) regulated significantly differentially expressed genes

To assess the compensation of chromosomes by the homoeologous tetra-chromosomes, we compared the number of expressed genes and the level of expressed genes in chromosome 7 between the NT stocks and CS. Here, we first used the ratios between the numbers of expressed genes in NT stocks and CS to show the contribution to the dosage compensation of genes in tetra-chromosomes. The scales were 0–1.0, 0–1.2, and 0–1.5 for the LEGs, MEGs, and HEGs, respectively (Fig. 3a). The ratios for LEGs in tetra-chromosomes is similar or less than the diploid chromosomes, while different for MEGs and HEGs. LEGs had a expressed gene number in the tetra-chromosomes of NT stocks similar to that in CS where the scale in tetra-chromosomes was less than 1 (the top of Fig. 3a), while there were more genes expressed in tetra-chromosomes of NT stocks among those mediumly and highly expressed genes than that in CS where the scales in the tetra-chromosomes were from 1 to 1.2 (the middle of Fig. 3a) and 1 to 1.5 (the bottom of Fig. 3a), respectively. Compared with CS, the differential gene expression patterns for LEGs, MEGs and HEGs were similar to the ratio patterns of expressed gene numbers between NT stocks and CS (Fig. 3c). The whole differential gene expression patterns for LEGs in tetra-chromosomes were similar to the diploid chromosomes in NT stocks, while these patterns for MEGs and HEGs were higher in tetra-chromosomes than in diploid chromosomes in NT stocks. Thus, The results suggested that the LEGs missing because of the nullisomic chromsomes were adequately compensated by the tetrasomic chromosomes of the NT stocks. By contrast, the MEGs and HEGs had an obvious strong variation either in the expressed gene number or the gene expression level in the tetra-homoeologous chromosomes compared with those in the diploid-homoeologous ones (Fig. 3a and c). A positive correlation was detected between the gene expression level and the number of additionally expressed genes (*r*^*2*^ = 0.9211, *P-value* = 3.05e-10; Fig. 3b), i.e., additional genes were expressed at the high level of gene expression, which indicated that the HEGs had a stronger compensation response than that of the LEGs in the NT stocks.

To investigate the significantly differentially expressed genes (SDEGs) in genome-wide level, we defined them in the NT stocks, relative to those in CS, using the DEGseq program with an FDR less than 0.001. A total of ~ 4000–5000 genes were SDEGs (Additional files 5, 6, 7, 8, 9 and 10). The up-regulated SDEGs with medium and high expression levels were also much more abundant than the lowly expressed SDEGs (Fig. 3e and Additional file 11: Figure S3A-C1, and Additional file 12: Table S3), whereas for the down-regulated SDEGs, most of them were the LEGs (Fig. 3f and Additional file 11: Figure S3A-C, and Additional file 12: Table S3). The MEGs occurred in the highest proportion followed by the HEGs (Fig. 3e, and Additional file 12: Table S3) in the up-regulated SDEGs, while the LEGs had the highest frequency and MEGs and HEGs with similar frequencies in the down-regulated SDEGs (Fig. 3f, and Additional file 12: Table S3).

For MEGs and HEGs, the SDEGs were significantly enriched in the tetra-chromosomes with all *P-values* less than 2.2e-12 by Fisher’s exact test, whereas for the LEGs, the proportion of SDEGs on the tetra-chromosomes was similar to that of CS (Fig. 3d). The stronger response of the MEGs and HEGs than that of the LEGs suggested that the corresponding increase of gene expressed dosage after the local chromosome duplication might balance the gene regulation network, which could directly compensate for the null-function by the deletion of their homoeologous chromosomes in the regulation network. As the dosage-sensitive genes in the local chromosome duplication, the MEGs and HEGs contributed to the gene dosage compensation for the whole chromosome deletion, whereas the LEGs did not make such a contribution.

### Differential expression of genes is organised in the chromosome domains

**Fig. 4:**
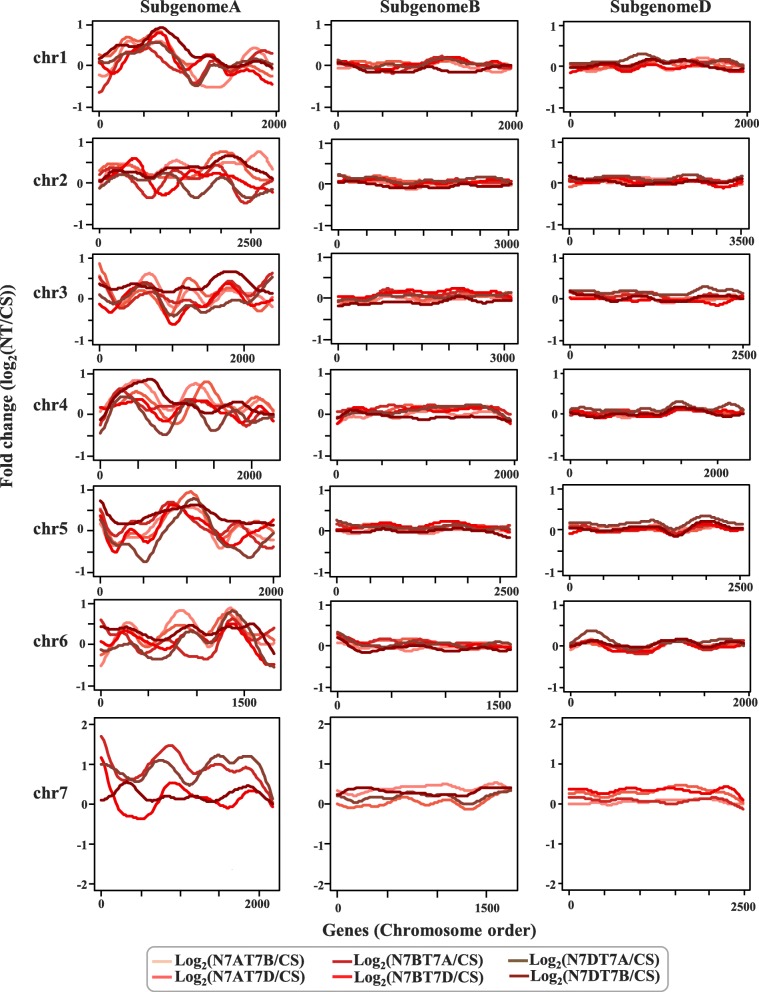
Fold changes (log_2_(NT/CS)) of gene expression were organized in chromosomal domains. The profile comparison of the differentially expressed genes between the NT stocks and CS along the wheat chromosomes

For the general transcriptomic changes, using the genome zipper available in the IWGSC website, we detected the genome-wide distribution of differentially expressed genes between the NT stocks and CS along the chromosomes with the window size of 50 genes. Subgenome A had the most variation in the gene expression profile at different chromosome domains compared with subgenomes B and D (Fig. 4). Furthermore, domain preference for the fold-change of expressed genes was observed along the chromosome, which had similar preferred profiles among the six NT stocks (Fig. 4). This comparison revealed that the well-defined chromosome domains had the differentially expressed gene profiles. These data showed that the gene expression patterns between NT stocks and CS were biased and organized in the chromosome domains.

Additionally, besides for chromosome 7, most of the SDEGs distributed on chromosomes 1–6 were either up-regulated or down-regulated genes (Additional file 11: Figure S3D-F), which indicated that the change in the expression level of some genes affected their upstream or downstream genes in the related pathway. The whole gene regulation interaction network buffered the gene imbalance by the response to the dosage effect of some genes.

Besides for the preferred distribution of the mediumly and highly expressed SDEGs on the tetra-chromosomes (Fig. 3d), the lowly, mediumly, and highly expressed SDEGs also preferred to locate in chromosomes 2D and 3D with all *P-values* less than 0.05 by Fisher’s exact test (Additional file 11: Figure S3G). The detailed distribution of the SDEGs on the chromosomes is shown in Additional file 13: Figure S4, which indicated that more interactions might occur at the chromosome level or the gene regulation level between chromosome 7 and chromosome 2 or 3.

### Response of the homoeologous genes in the NT stocks

**Fig. 5:**
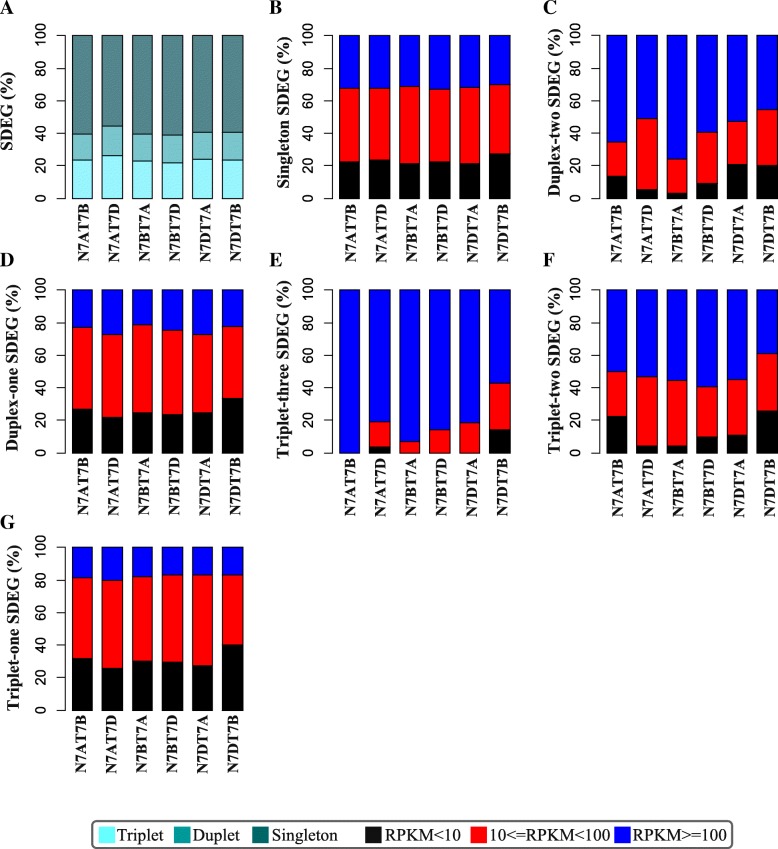
The categories of the SDEGs. **a** Distribution of the SDEGs for the three categories including triplet genes, duplet genes, and singleton genes. The light cadet blue, cadet blue, and dark cadet blue represented the triplet genes, duplet genes, and singleton genes, respectively; **b** Distribution of singleton genes for the lowly (black rectangle), mediumly (red rectangle), and highly expressed genes (dark rectangle); **c**-**d** Distribution of duplet genes with two (**c**) or one (**d**) homoeologous SDEGs for the low, medium and high expression level; **e**-**g** Distribution of triplet genes with three (**e**), or two (**f**) or one (**g**) homoeologous SDEGs for the low, medium and high expression level

The chromosome structure variance triggered the variation in morphology and the level of gene expression. The variation was concentrated in subgenome A and chromosomes 7A, 7B, 7D, 2D and 3D, as described in the above section. To understand the influence of the homoeologous genes in the NT stocks, we first divided the genes into three categories: triplet genes, three homoeologous genes retained in all of the three subgenomes; duplet genes, one homoeologous gene lost and the other two retained in both of the two subgenomes, and singleton genes, only one homoeologous gene retained. The distribution of SDEGs within the three types of genes showed that most of the SDEGs (55–60%; Fig. 5a) were classified as singleton genes.

For the singleton genes, most of them were MEGs followed by HEGs and LEGs (Fig. 5b). For the duplet genes with two SDEGs (Duplet-two SDEGs), most of them were HEGs followed by MEGs (Fig. 5c), whereas only a few of them were LEGs; however, for the duplet genes with only one SDEG (duplet-one SDEGs), similar to the singleton genes, most of them were MEGs followed by HEGs and LEGs (Fig. 5d).

For the three copies of triplet genes with three or two SDEGs (triplet-three or triplet-two SDEGs), almost all of them were HEGs followed by MEGs and only a few of them were LEGs (Fig. 5e-f). However, for the triplet genes with only one SDEG (triplet-one SDEGs), the distribution of the gene expression level was very similar to that of the duplet-one SDEGs or the singleton genes. This result indicated that the lowly expressed duplet-one SDEGs, triplet-one SDEGs and the singleton genes might have less important functions, whereas the duplet-two, triplet-three, and triplet-two SDEGs with highly expressed level might be associated with important of biological functions.

### Perturbation of the biological function in the NT stocks

For aneuploid organisms, including fungi such as yeast, animals such as mouse and human, and plants such as *Arabidopsis*, the pattern of high expression level of genes affects similar and conserved cellular pathways. Genes involved in a stress response are consistently up-regulated, whereas those genes associated with the cell cycle and cell proliferation are down-regulated [5]. To understand the function of SDEGs in the NT stocks, we performed GO enrichment analysis using the BINGO program with reference to the CS genome (Version 2). Although without replicates of the RNA-seq dataset because the phenotypes between N7AT7B and N7AT7D, N7BT7A and N7BT7D, and N7DT7A and N7DT7B were similar, we used the overlapped SDEGs between N7AT7B and N7AT7D, N7BT7A and N7BT7D, and N7DT7A and N7DT7B as the N7A, N7B, and N7D SDEGs, respectively (Additional file 14: Figure S5). For the down-regulated common SDEGs, 536 LEGs, 150 MEGs, and 220 HEGs were identified in N7A, 447 LEGs, 111 MEGs, and 213 HEGs in N7B, and 513 LEGs, 143 MEGs, and 191 HEGs in N7D. For the up-regulated common SDEGs, 557 MEGs and 436 HEGs were identified in N7A, 472 MEGs and 269 HEGs in N7B, and 520 MEGs and 357 HEGs in N7D.

**Fig. 6:**
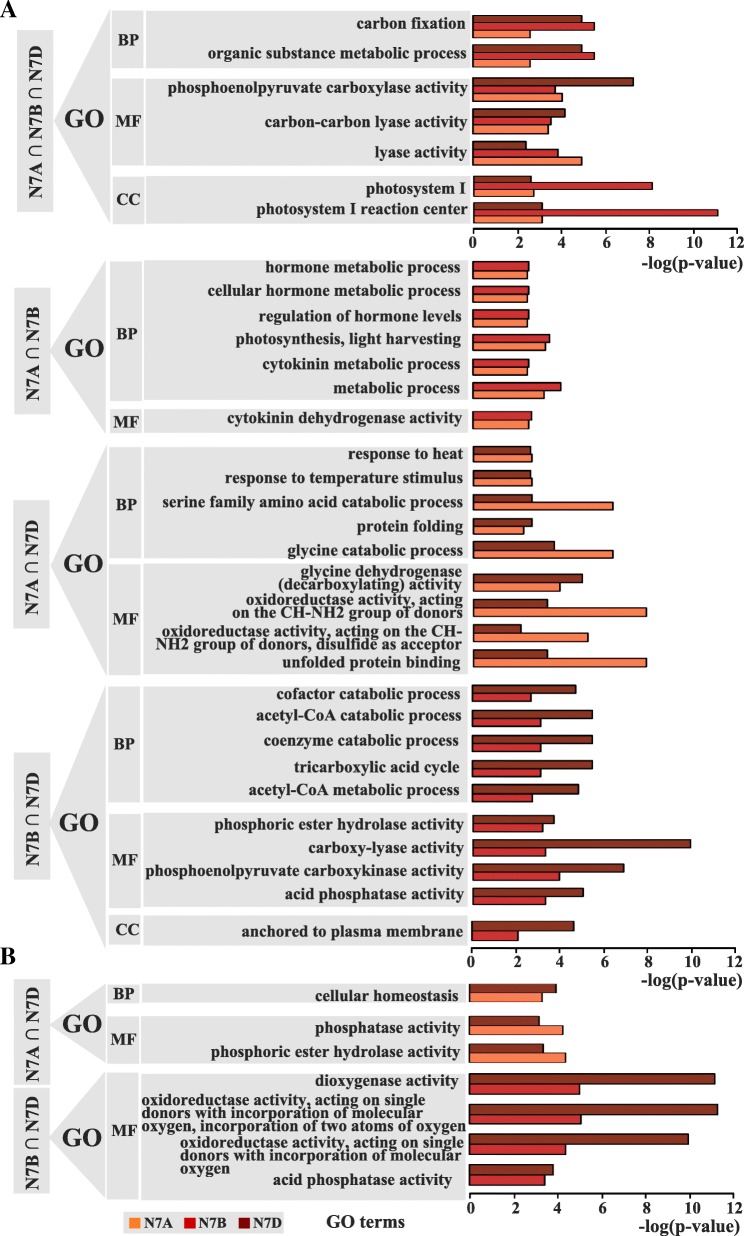
The GO enrichment analysis in the down-regulated (**a**) and up-regulated (**b**) genes overlapped among N7A (N7AT7B∩N7AT7D), N7B (N7BT7A∩N7BT7D), and N7D (N7DT7A∩N7DT7B) referred to CS

Then, using the common SDEGs, we performed GO enrichment analysis as shown in Fig. 6 and Additional file 15: Figure S6. GO term analysis of the down-regulated genes shared among N7A, N7B, and N7D revealed that most of the enriched categories with FDR < 0.05 were carbon metabolic processes such as carbon fixation, carbon-carbon lyase activity, and photosystem metabolic process such as photosystem I and photosystem I reaction center (Fig. 6a). For the down-regulated SDEGs between N7A and N7B, most of the GO terms were enriched in the hormone metabolic process (Fig. 6b). For the down-regulated SDEGs between N7A and N7D, most of the GO terms were enriched in the stress response such as heat and temperature stimuli (Fig. 6b). For the down-regulated SDEGs between N7B and N7D, most of the GO terms were enriched in the catabolic process such as cofactor, coenzyme, and acetyl-CoA (Fig. 6b). For the up-regulated genes between N7A and N7D, the GO terms of SDEGs were enriched in the cellular homoeostasis (Fig. 6c); between N7B and N7D, the GO terms of SDEGs were enriched in the oxidoreductase activity (Fig. 6c). However, for the up-regulated genes, no GO category was enriched among N7A, N7B, and N7D or between N7A and N7B. Additionally, no GO term was particularly enriched in N7A or N7B, whereas for N7D, the up-regulated SDEGs were particularly enriched in the photosynthesis process and included photosynthetic electron transport in photosystem I, photosynthetic electron transport chain, and light reaction. Furthermore, more of the SDEGs were located in the photosynthetic membrane and thylakoid membrane than in other structures (Fig. 6 and Additional file 15: Figure S6). According to the above description, the SDEGs in the NT stocks for group 7 primarily affected the photosynthesis process, carbon fixation, and the stress response, which might explain the final deficient phenotypic traits that included reductions in plant height and spike number and infertility of the top spikelets, which may contribute to the phenotype of fewer kernels in per spike.

In NT stocks, there also were many NT-specific SDEGs in each nullisomic stocks (Additional file 14: Figure S5). In N7A stocks, 943 and 1488 N7AT7B-specific SDEGs, and 1253 and 2128 N7AT7D-specific SDEGs, were up- and down-regulated, respectively. In N7B stocks, 1224 and 1677 N7BT7A-specific SDEGs, and 1002 and 1846 N7BT7D-specific SDEGs, were up- and down-regulated, respectively. In N7D stocks, 1222 and 2066 N7AT7B-specific SDEGs, and 1465 and 1585 N7AT7D-specific SDEGs, were up- and down-regulated, respectively. The up-regulated NT-specific SDEGs were also much more abundant than the down-regulated ones. Then, using the NT-specific SDEGs, we performed GO enrichment analysis as shown in Additional files 16, 17, 18, 19, 20 and 21. GO term analysis of the down- and up-regulated genes for the NT-specific genes, revealed that some of GO-terms were shared for the NT-specific SDEGs in N7A, N7B and N7D stocks. In N7A stocks, there were no common GO terms enriched in N7AT7B-specific and N7AT7D-specific SDEGs (Additional files 16 and 17). In N7B stocks, the SDEGs with the same GO terms such as catabolic and metabolic processes of glycine and serine family, had the reverse expression patterns in N7BT7A and N7BT7D stocks (Additional files 18 and 19). In N7D stocks, small molecule, glucose, alcohol, sulphur, carbohydrate, hexose, lipid metabolic or catabolic process, and the glycolysis were common enriched for the down-regulated SDEGs. Whereas the GO terms of the photosynthesis and light harvesting or reaction were also both enriched with the reverse expression patterns for the SDEGs in N7DT7A and N7DT7B stocks (Additional files 20 and 21). It may indicated that specific enriched gene functions may less contribute to the phenotypes than the common ones since the similar phenotypes in the nullisomic stocks.

For the variation of gene expression in the NT stocks compared with that in CS, most of the genes were from the other chromosomes, besides chromosome 7A, 7B and 7D, as described above. Furthermore, the variation in gene expression was localized to chromosome 2D, besides chromosome 7A, 7B and 7D. It suggested that the gene expression change in chromosome 7A, 7B and 7D triggered an effect on their interaction genes. Additionally, the differentially expressed genes derived from different chromosomes enriched in the same GO terms suggested a gene interaction network among these genes. When the change occurs with a gene located in a core position of the gene regulation network, the whole gene regulation network will be imbalanced as a result of the imbalance at the level of gene expression.

## Discussion

### Chromosome karyotypes determine the deficient phenotypes

**Fig. 7:**
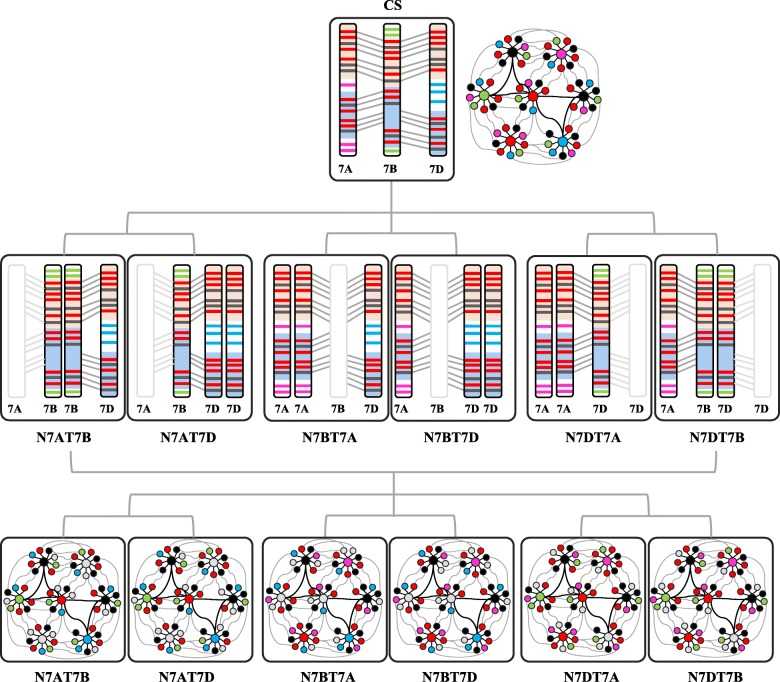
The Module of gene partial dosage compensation due to the asymmetrical gene distribution (the top and middle panels) in the gene regulation network (the top and bottom panels) for the NT stocks of group 7. Red circles and rectangles represented the triplet and duplet genes; Purple, green and blue ones represented for the singleton gene on the chromosome 7A, 7B and 7D respectively; The grey ones represented the null-homoeologs; The black ones represented the genes on the non-chromosome 7

A polyploidisation event occurs at least once in the evolution history of most angiosperm species [19]. The genome doubles, derives the gene dosage effect of polyploidisation or the neofunctionalisation of the homoeologous genes, which leads to heterosis with changes in chromatin modification and the gene expression regulatory network. It improves the robust adaptation of plants to environmental abiotic and biotic stresses [20]. In allopolyploid cotton [21], mesopolyploid *Brassica rapa* [22], synthetic allotetraploid *Arabidopsis* [23], and the paleoploid maize genome, the gene expression variations indicated that one genome was preferentially more transcriptionally activated than the others. For the allohexaploid wheat with approximately ten thousand years of evolutionary history of domestication, most of the genes globally showed an additive level of expression of the three subgenomes [8, 13, 24]. For the NT stocks of hexaploid wheat, the other homoeologous chromosomes largely buffered the gene dosage for any one set of null-homoeologous chromosomes. The polyploid NT stocks with similar chromosome karyotypes showed similar dosage effects. Despite of it, the cell type- and stage-dependent genome dominance showed transcriptionally actively chromosomal regions and asymmetric expression [13, 25]. With approximately ten thousand years of evolutionary history of domestication for hexaploid wheat, a few of homoeologous genes showed asymmetric distribution, functional diversity and nonadditive level of expression. For the asymmetrical distribution in the three sets of the wheat subgenomes, approximately half of the coding genes were singleton genes with only one copy of a homoeolog, while the others with two or three homoeologous copies (Fig. 7). For gene that has only one functional homoeolog on one subgenome (also called singleton), once this homoeolog was removed in the NT stocks, its function can’t be compensated by the other two subgenomes which carried non-functional homoeologs. In such conditions, phenotypic changes were more frequently observed than those who have functional homoeologs on the other two subgenomes. We also provided examples of such subgenome replacement on phenotype changes (Fig. 1). For example, the ***TaGH3*** (*Gretchen Hagen3*) families have 31 homoeologous copies totally in hexaploid wheat. Of these copies, 27 are distributed in the subgenomes A and B and only four are located in subgenome D. *TaGH3.1* has two alleles on chromosome 3A, with none in subgenomes B and D. It showed asymmetrical distribution in the three subgenomes on common wheat [26].

In the hexaploid wheat, the homoeoalleles globally showed additive expression but with local nonadditive asymmetric expression. However, the local asymmetric expression may contribute to excellent agronomic traits. For example, change in the expression pattern of the D homoeoallele *HKT1;5* (*High-Affinity K+ Transporter 1;5*) increases the salt tolerance of hexaploid wheat [27]. For the altered function of three homoeologs, some genes showed null-functionalisation as redundant copies or in subfunctionalisation. For example, the three wheat homoeologs of wheat SEPALLATA (*WSEP*) and the homologs of *OsMADS45* (*MCM1, AG, DEFA and SRF 45*) in rice showed similar genomic structures and expression profiles. By contrast, the three homoeologs of wheat *LEAFY HULL STERILE1* (*WLHS1*) and the homologs of *OsMAD1* in rice showed genetic and epigenetic alterations. WLHS1-A, because of structural alteration, had no apparent function; WLHS-B, because of cytosine methylation, was also silenced; only WLHS-D had functions in hexaploid wheat [28]. For the *Q* gene, a member of the *AP2* transcription factors, also with three homoallele copies, only the 5*AQ* as the dominant allele with hyper-functionalisation played a primary role in conferring domestication-related traits [29, 30]. Thus, the gene alleles with function partitioning diversified after the hexa-polyploidisation domestications during approximately ten thousand years.

Additionally, strong positive selection fixed many genes in the early breeding in the domestication of wheat [31]. As examples, consider *TaSus2* (*Sucrose synthase 2*), *TaGW2*, *TaTEF* (*transcript elongation factor*) and *TaGS5*. *TaSus2* could be active in endosperm development. In the wheat accession, two haplotypes, Hap-H and Hap-L, in *TaSus2B* and the Hap-A of *TaSus2A* were significantly associated with the thousand kernel weight (TKW), whereas no diversity was found in *TaSus2D* [32]. *TaGW2-6B* has a stronger influence than *TaGW2-6A* on TKW (Thousand Kernel Weight) [31], and *TaTEF-7A*, as a functional regulatory factor for grain number in per spike, had a higher level of expression than that of *TaTEF-7B* and *TaTEF-7D* [33]. The two alleles *TaGS5-3A* and *TaGS5-3A-T* with higher enzymatic activity than that of *TaGS5-3A-G* were significantly correlated with larger grain size and higher TKW [34].

Overall, besides the variations in the three sets of subgenomes derived from their progenitors, for most of the genome regions without partitioning that remained with three redundant gene copies and additive expression profiles, dosage compensation could buffer the null-homoeologous chromosomes. However, the asymmetric gene distribution in the subgenome, functionalisation diversity, including null−/sub−/neo-functionalisation, for the homoeologous copies and expression variance of homoeoalleles, with domestication favouring the homoeoalleles, contributed to the subgenome plasticity. The similarity and heterogeneity of the three subgenomes determined the imperfect dosage compensation for the NT stocks, which led to the defective phenotypes in the NT stocks. Some differentially expressed genes were important transcript factors, such as *WRKY*, *NAC* (*NAM, ATAF, and CUC*), *MYB*, *ARF* (*Auxin Response Factors*), *AP2/ERF* (*AP2-like ethylene-responsive transcription factor*), *GATA*, *NFYA* (*Nuclear transcription factor Y subunit A*), *SPB* (*Squamosa promoter binding like protein*), *WD*, and *MADS*-box, and some of these might be singleton or functionalisation diversity homoeologs. For these deleted genes involved in the interaction of multiple genes, dysregulation of one gene will affect the entire balance of pathway regulation (Fig. 7).

### Advantages and disadvantages to the organism from local large-scale genome duplication or deletion

The gene balance hypothesis posits that altering the stoichiometry of members of multi-subunit complexes will affect the function of the whole as a result of the kinetics and mode of assembly [35]. Because the diploidisation process allows retention of multiple interacting components of a complex, such as occurred in polyploid wheat and cotton after whole-genome duplication (WGD) events, the gene dosage balance is not perturbed [36]. By contrast, the dosage of a varied chromosome arm can affect the expression of multiple genes located throughout the genome, which results in both positive and negative correlations with gene expression. Concerning the relative expression level, the slight alterations of transcription factors or other regulatory proteins in the affected chromosomal region might affect the expression of multiple genes throughout the genome, causing massive alterations in gene network functioning due to complex gene interactions.

These kinds of perturbations of gene networks can lead to positive results. For example, the wheat-rye 1BL•1RS translocation line conferred superior genes, including those for biotic and abiotic stress tolerance, high yield potential, and wide adaptability to different environments, which contribute to worldwide production [37–41]. The wheat-rye 4R and 6R chromosome translocation lines showed strong resistance to powdery mildew [42, 43]. For *Psathyrostachys huashanica* Keng, all of the 2 N, 3 N, 4 N and 7 N chromosome addition stocks carried new leaf rust resistance genes and increased tiller numbers and had a superior number of florets and grains per spike [24, 44–46]. Wheat-*Thinopyrum* alien addition disomic line germplasm SN6306 was also observed with resistance against powdery mildew using transcriptome analysis [47]. A new wheat-*Aegilops biuncialis* addition stock improved quality-associated HMW glutenin subunits [48]. Aluminium tolerance increased in wheat derived from the addition of chromosomes from the wild relative *Leymus racemosus* [49]. This suggests that polyploid organisms have a much higher tolerance for an increase in chromosomes than those of diploids.

However, for the deletion stocks, the wheat always showed the defective type of phenotype in morphological, physiological, and biochemical traits [50]. For example, one trait was male sterility, such as occurred in part of 1DL, 4BS, 5AS, and 5BS deletion stocks, whereas part of the 2AL deletion stock was highly sterile in both sexes. Some of them had reduced selfed-fertility, such as part of the 2BL, 5BL and 6BL deletion stocks. Both sterility and reduced fertility decrease the seeds in per spike, which also indicates that the genes associated with fertility are located in those chromosome arms. Additionally, the shape of the spikes were also changed in the deletion stocks compared with that of CS, such as the awned spike in 6BL-15, the short spike in 6BL-14, and the full speltoid spike in 5AL-7, suggesting the morphological defect was due to the chromosome deletion. However, in the hexaploid wheat, the chromosome deletion stocks always caused harm to the organism because of the adverse mutant phenotypes. In maize, the hypoploid maize embryo with a single dosage of some chromosome arms also showed more deficient phenotypes than those of the hyperploid embryo lines with three dosages of some chromosome arms for leaf width and length, plant height, primary ear height, internode length, stalk circumference, and tassel branch number [51].

Organisms have much less tolerance for the loss of chromosomes than for the additional ones. Burns and Kacser proposed that a slight dosage change of genes encoding most metabolic enzymes would not affect the fitness. The flux of the pathway dictates the enzyme activity, and a 0.5-fold change in enzyme concentration has minimal effects [52, 53]. It explains that the lines with additional chromosomes show stronger adaptation to stressed environments than deletion stocks. For the NT stocks, as either addition or deletion stocks, more genes were up-regulated than down-regulated. The tetra-chromosomes mostly compensated for the null-chromosomes in the level of gene expression, even with the local bias. Therefore, the increased dosage in the hexaploid NT stocks might slightly alter the balance of the gene network, whereas the reduction in gene dosage level disturbed the gene regulation pathway to some extent, which contributed to the deficiently phenotypic traits.

### Altered gene dosage contributed to the resistance to chromosome disturbance

Compared with the addition stocks with trisomy specific chromosomes and the deletion stocks with deleted chromosomes or arms, the phenotypes in the NT stocks had slight deficiencies. For aneuploidy, the enriched function for the differentially expressed genes always involved a response to stress [5, 54–58], whereas for the NT stocks, the enrichment also focused on the photosystem reaction centre (Fig. 6). The slight variation in the phenotype might be regulated by the variation of gene expression in response to the stress of chromosome alteration. First, the up-regulated genes might contribute to the resistance to the chromosome disturbance. In the NT stocks, more genes were up-regulated than down-regulated (Fig. 2c), and the altered expression of genes might be a response to the chromosome replacement events. Second, the NT stocks increased the number of expressed genes in response to the genome variation, which was positively correlated with the level of gene expression (Fig. 3a-b). Third, the biased expression profile throughout the whole genome might also contribute to the response to chromosome replacement (Fig. 3d, Additional file 11: Figure S3G and Fig. 4), similar to the response found in Down’s syndrome [59]. Furthermore, many triplet and duplet genes were significantly differentially expressed and most of which were high and medium expression genes (Fig. 3b-g). The triplet or duplet genes with high expression level in the hexaploid *Brassica rapa* also have important biological functions and were enriched in transcriptional regulation, ribosomes, response to stimuli, cell organisation, and transporter functions [22, 60]. The retention of multiple components of a complex can resist the diploidisation following the polyploidisation, which maintains the genome balance [36]. The triplet and duplet genes with important biological functions might have an increased contribution to the resistance of the genome disturbance with the substitution of homoeologs (Fig. 7). The genes from the tetra-chromosomes and triplet-three, triplet-two, or duplet-two homoeologs or the paleo-duplicated pairs can all buffer the gene dosage-variation, which might contribute to the slight phenotype mutation after large-scale variation of chromosomes.

## Conclusions

In summary, our results provided the first transcriptome profiles in the classical genetic materials of the NT stocks in hexaploid wheat. The differential gene expression in the NT stocks revealed the roles of gene dosage effect in response to the large-scale variation of chromosomes. Further study of the dosage-effect mechanism for gene deletion and compensation should improve our understanding of the function of subgenomes in modern polyploid plants.

## Additional files

Additional file 1: **Figure S1.** FISH on the metaphase NT stocks for group 7 using Oligo-pTa535-1 (red) and Oligo-pSc119.2 (green) as probes. A-F represented the N7AT7B, N7AT7D, N7BT7A, N7BT7D, N7DT7A, and N7DT7B, respectively. (PDF 143 kb)

Additional file 2: **Figure S2.** The flow chart of RNA-seq data analysis on the NT stocks referred to CS genome. (PDF 21 kb)

Additional file 3: **Table S1.** The mapping information of the transcriptomes with only one hit loci referred to the CS genome in the NT stocks for group 7 and CS. (XLS 20 kb)

Additional file 4: **Table S2.** The expressed genes in the NT stocks for group 7 and CS. (XLS 25 kb)

Additional file 5: Significantly differentially expressed genes with FDR less than 0.001 in N7AT7B stock compared with CS. (XLSX 416 kb)

Additional file 6: Significantly differentially expressed genes with FDR less than 0.001 in N7AT7D stock compared with CS. (XLSX 500 kb)

Additional file 7: Significantly differentially expressed genes with FDR less than 0.001 in N7BT7A stock compared with CS. (XLSX 416 kb)

Additional file 8: Significantly differentially expressed genes with FDR less than 0.001 in N7BT7D stock compared with CS. (XLSX 412 kb)

Additional file 9: Significantly differentially expressed genes with FDR less than 0.001 in N7DT7A stock compared with CS. (XLSX 475 kb)

Additional file 10: Significantly differentially expressed genes with FDR less than 0.001 in N7DT7B stock compared with CS. (XLSX 451 kb)

Additional file 11: **Figure S3.** The number of the up- and down-regulated genes for the lowly (A), mediumly (B), and highly (C) expressed genes. (D-F) The distribution of SDEGs in the chromosome 1–6 and chromosome 7 for the lowly (D), mediumly (E) and highly (F) expressed genes. (G) The preference of proportion distribution of SDEGs on the chromosome 2D and 3D in the NT stocks compared with that in CS. (PDF 51 kb)

Additional file 12: **Table S3.** The significantly differentially expressed genes in the NT stocks for group 7 compared with CS. Chrs, chromosomes. (XLS 24 kb)

Additional file 13: **Figure S4.** The distribution of significantly differentially expressed genes along the chromosomes. (PDF 57 kb)

Additional file 14: **Figure S5.** The overlapped up- and down-regulated genes in N7A (N7AT7B ∩ N7AT7D), N7B (N7BT7A ∩ N7BT7D), and N7D (N7DT7A ∩ N7DT7B) stocks. (PDF 105 kb)

Additional file 15: **Figure S6.** The GO enrichment analysis for the up-regulated genes in N7D (N7DT7A ∩ N7DT7B) stocks referred to CS. (PDF 52 kb)

Additional file 16: The GO enrichment for the N7AT7B-specific down- or up-regulated expressed genes in N7A stocks. (XLS 40 kb)

Additional file 17: The GO enrichment for the N7AT7D-specific down- or up-regulated expressed genes in N7A stocks. (XLS 250 kb)

Additional file 18: The GO enrichment for the N7BT7A-specific down- or up-regulated expressed genes in N7B stocks. (XLS 66 kb)

Additional file 19: The GO enrichment for the N7BT7D-specific down- or up-regulated expressed genes in N7B stocks. (XLS 52 kb)

Additional file 20: The GO enrichment for the N7DT7A-specific down- or up-regulated expressed genes in N7D stocks. (XLS 87 kb)

Additional file 21: The GO enrichment for the N7DT7B-specific down- or up-regulated expressed genes in N7D stocks. (XLS 57 kb)

## Abbreviations

AP2/ERF: AP2-like ethylene-responsive transcription factor
ARF: Auxin Response Factors
CS: Chinese Spring
FDR: False discovery rate
FISH: Fluorescence In Situ Hybridisation
GH3: Gretchen Hagen3
GO: Gene Ontology
HEG: Highly expressed gene
HKT1;5: High-Affinity K+ Transporter 1;5
LEG: Lowly expressed gene
MADS: MCM1, AG, DEFA and SRF
MEG: Mediumly expressed gene
NAC: NAM, ATAF, and CUC
NFYA: Nuclear transcription factor Y subunit A
Nulli-Tetra/NT: Nullisomic-tetrasomic
SDEG: Significantly differentially expressed gene
SPB: Squamosa promoter binding like protein
TaSus2: Sucrose synthase 2
TaTEF: Transcript elongation factor
TKW: Thousand kernel weight
WGD: Whole genome duplication
WGRC: Wheat Genetic Resource Center
WLHS1: Wheat LEAFY HULL STERILE1
WSEP: Wheat SEPALLATA
