# Supplementary figures and images for: The genome-wide transcriptional consequences of the nullisomic-tetrasomic stocks for homoeologous group 7 in bread wheat
Source: BMC Genomics. 2019 Jan 10;20:29. doi: 10.1186/s12864-018-5421-3 (PMC6327598; doi:10.1186/s12864-018-5421-3)

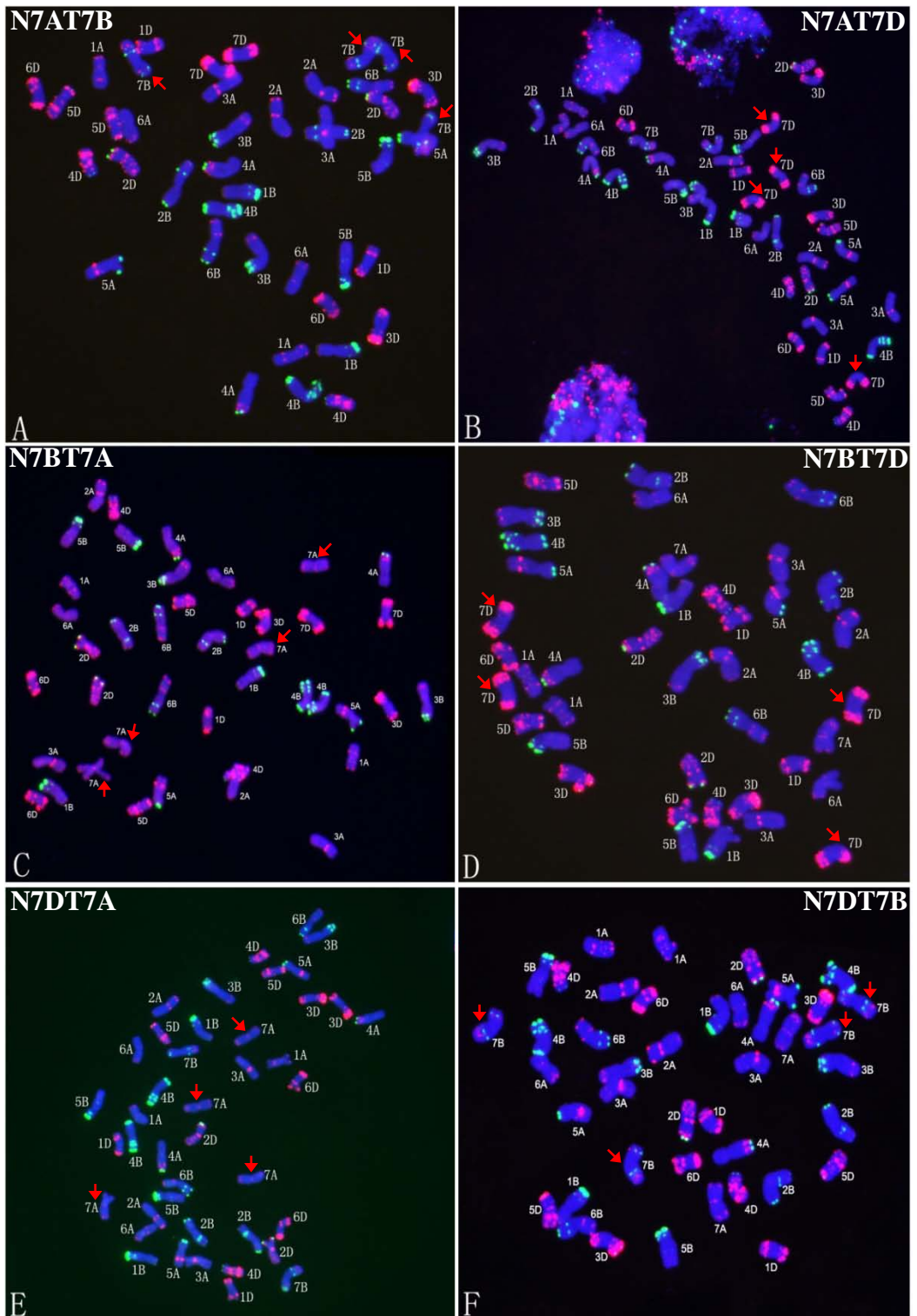

**Figure S1**

Supplement: Supplementary file 1 — Figure S1. FISH on the metaphase NT stocks for group 7 using Oligo-pTa535-1 (red) and Oligo-pSc119.2 (green) as probes. A-F represented the N7AT7B, N7AT7D, N7BT7A, N7BT7D, N7DT7A, and N7DT7B, respectively. (PDF 143 kb) [file 12864_2018_5421_MOESM1_ESM.pdf]

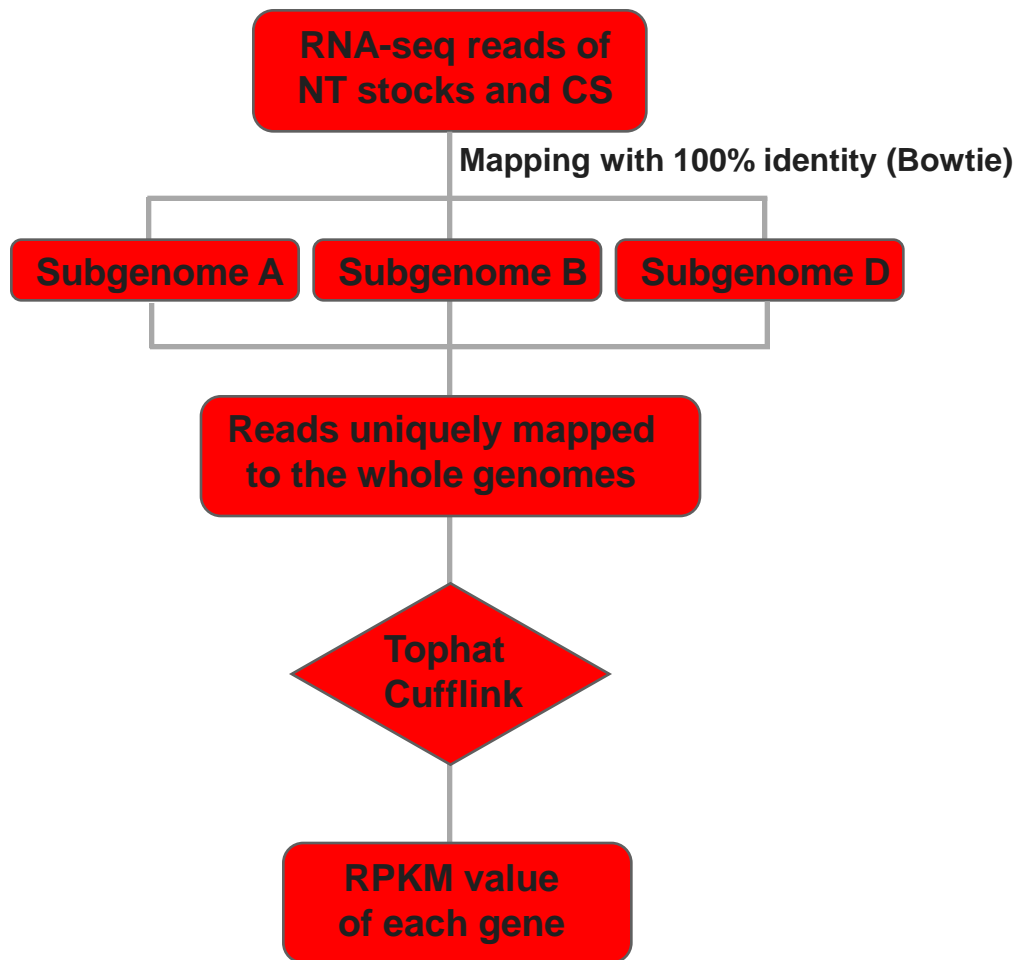

**Figure S2**

Supplement: Supplementary file 2 — Figure S2. The flow chart of RNA-seq data analysis on the NT stocks referred to CS genome. (PDF 21 kb) [file 12864_2018_5421_MOESM2_ESM.pdf]

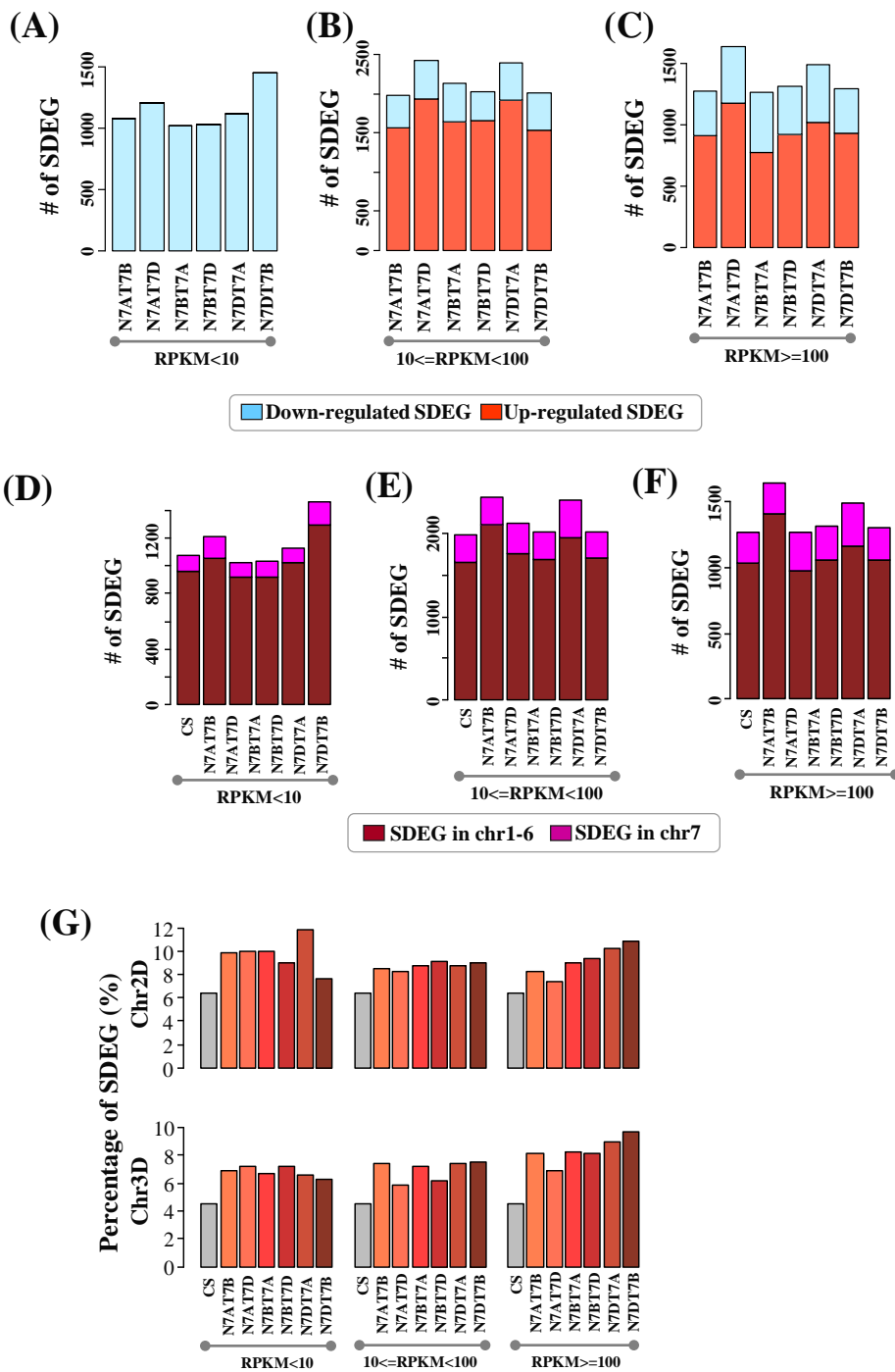

Supplement: Supplementary file 11 — Figure S3. The number of the up- and down-regulated genes for the lowly (A), mediumly (B), and highly (C) expressed genes. (D-F) The distribution of SDEGs in the chromosome 1–6 and chromosome 7 for the lowly (D), mediumly (E) and highly (F) expressed genes. (G) The preference of proportion distribution of SDEGs on the chromosome 2D and 3D in the NT stocks compared with that in CS. (PDF 51 kb) [file 12864_2018_5421_MOESM11_ESM.pdf]

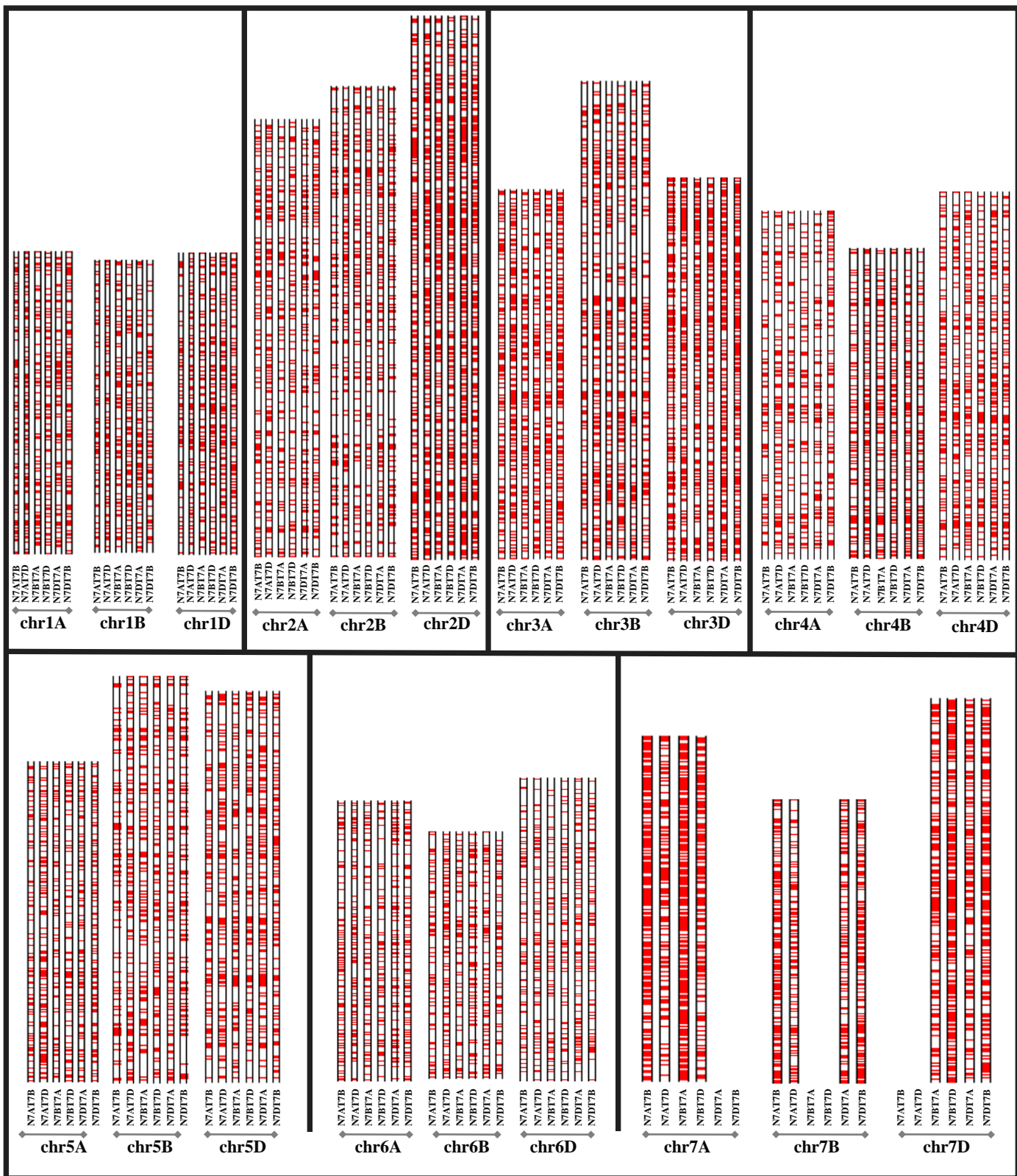

Figure S4

Supplement: Supplementary file 13 — Figure S4. The distribution of significantly differentially expressed genes along the chromosomes. (PDF 57 kb) [file 12864_2018_5421_MOESM13_ESM.pdf]

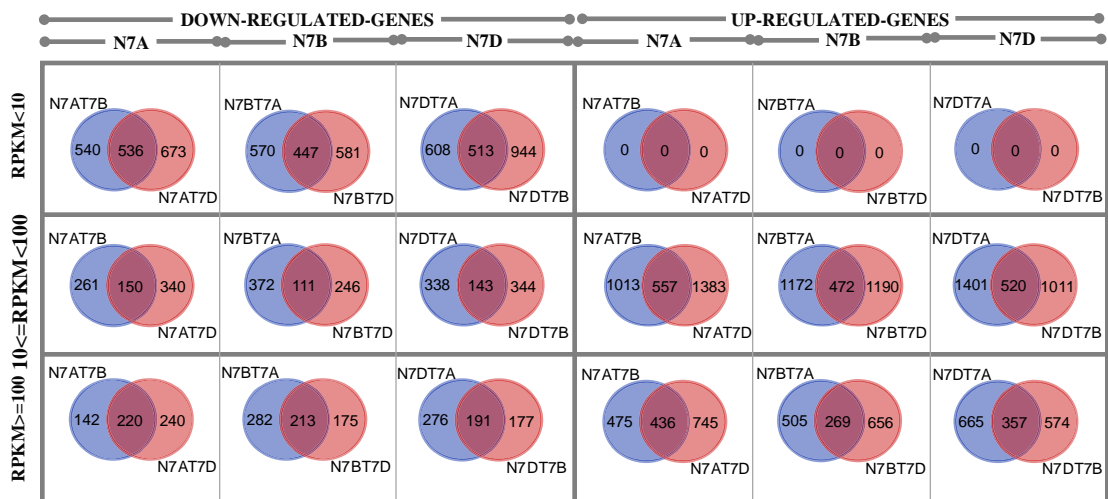

**Figure S5**

Supplement: Supplementary file 14 — Figure S5. The overlapped up- and down-regulated genes in N7A (N7AT7B ∩ N7AT7D), N7B (N7BT7A ∩ N7BT7D), and N7D (N7DT7A ∩ N7DT7B) stocks. (PDF 105 kb) [file 12864_2018_5421_MOESM14_ESM.pdf]

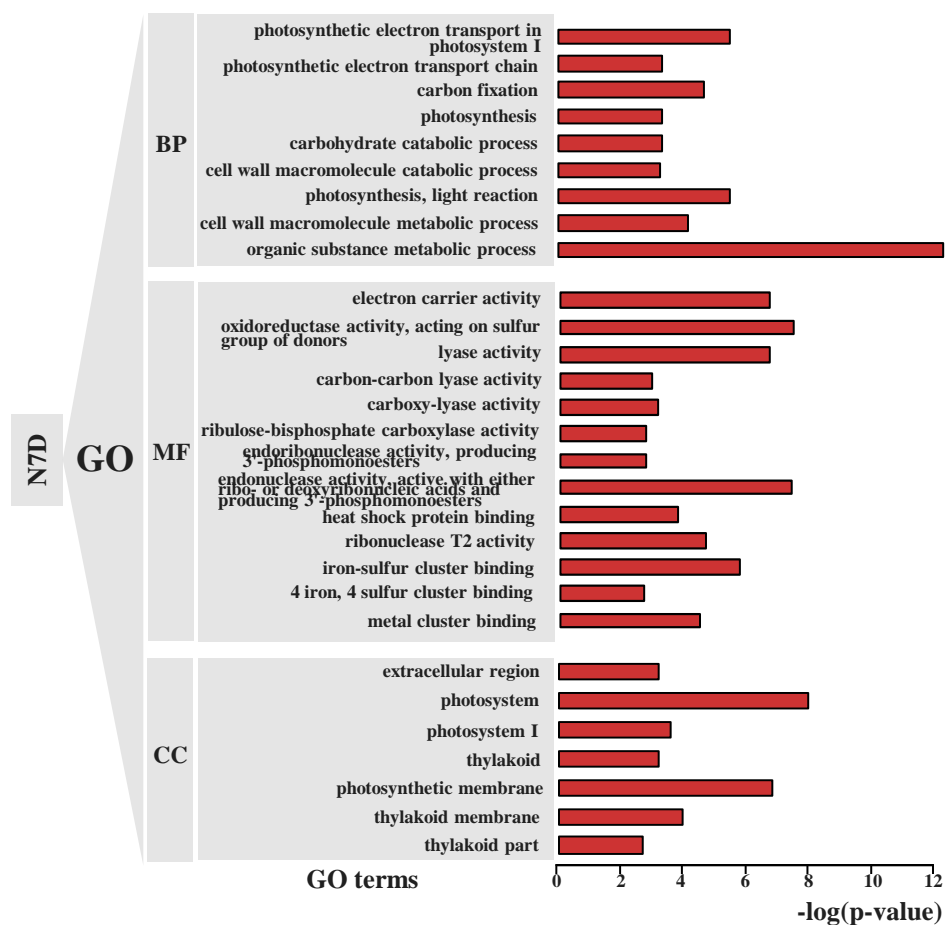

**Figure S6**

Supplement: Supplementary file 15 — Figure S6. The GO enrichment analysis for the up-regulated genes in N7D (N7DT7A ∩ N7DT7B) stocks referred to CS. (PDF 52 kb) [file 12864_2018_5421_MOESM15_ESM.pdf]
